# Spacing effects on flows around two square cylinders in staggered arrangement via LBM

**DOI:** 10.1038/s41598-024-66895-0

**Published:** 2024-08-05

**Authors:** Ahmed Refaie Ali, Waqas Sarwar Abbasi, Bakhtawar Bibi, Hamid Rahman, Shams Ul Islam, Afraz Hussain Majeed, Irshad Ahmad

**Affiliations:** 1 Department of Mathematics and Computer Science, Faculty of Science, Menoufia University, Shebin El Kom, Menofia, 32511 Egypt; 2 Department of Mathematics, Air University, Islamabad, 44000 Pakistan; 3 Department of Mathematics & Statistics, Women University Swabi, Swabi, 23430 Pakistan; 4 Department of Mathematics, COMSATS University, Islamabad, 44000 Pakistan; 5 School of Energy and Power Engineering, Jiangsu University, Zhenjiang, 212013 China; 6 Department of Medical Rehabilitation Sciences, College of Applied Medical Sciences, King Khalid University, Abha, Saudi Arabia; 7 Department of Industrial Engineering, Università di Bologna, Alma Mater Studiorum, Italy

**Keywords:** Fluid flow, Cylinder, Vorticity, Simulations, Drag force, Lift force, Mechanical engineering, Applied mathematics, Computational science, Fluid dynamics, Information theory and computation, Statistical physics, thermodynamics and nonlinear dynamics

## Abstract

This study presents a computational analysis of fluid flow characteristics around two staggered arranged square cylinders using the Lattice Boltzmann Method (LBM). With Reynolds number (Re) fixed at 200, numerical simulations explore the influence of varying gap ratios (G) ranging from 0 to 10 times the cylinder size. Emphasis is placed on understanding the impact of cylinders spacing on flow structure mechanisms and induced forces. Investigation of fluid flow parameters includes vorticity behavior, pressure streamlines, and variations in drag and lift coefficients alongside the Strouhal number under different values of G. From the results, four distinct flow patterns emerge: single bluff body flow, flip flopping flow, modulated synchronized flow, and synchronized flow, each exhibiting unique characteristics. This study reveals the strong dependence of fluid forces on G, with low spacing values leading to complex vortex structures and fluctuating forces influenced by jet flow effects. At higher spacing values, proximity effects between cylinders diminish, resulting in a smoother periodic flow. The Strouhal number, average drag force and the rms values of drag and lift force coefficients vary abruptly at narrow gaps and become smooth at higher gap ratios. Unlike the tandem and side-by-side arrangements the staggered cylinders arrangement is found to have significant impact on the pressure variations around both cylinders. Overall, this research could contribute to a comprehensive understanding of staggered cylinder arrangements and their implications for engineering applications.

## Introduction

Flow around multiple bluff bodies plays a significant role in various real-life and engineering applications, including chimneys, cooling towers, high-rise buildings, electronic devices, and bridges. In such applications, the arrangement of multiple bodies with respect to fluid streams is crucial. This study focuses on the staggered arrangement, which significantly influences the flow structure, pressure, and flow-induced forces compared to its side-by-side or inline counterparts. Understanding the flow characteristics, such as drag forces and wake formation in staggered bluff bodies, enables engineers to optimize the design of civil structures like buildings and bridges, as well as heat exchangers, to enhance their performance and strength. Investigating the flow-induced forces on staggered cylinders may lead to modifications in structural design to minimize drag, optimize heat transfer, and improve overall performance. This knowledge is valuable in industries such as aerospace, automotive, civil engineering, and energy production, where fluid dynamics play a critical role in efficiency and safety.

Analyzing the crossflow features around staggered arrangements has attracted many research studies. Numerous experimental and numerical studies have investigated such flows, with many early investigations focusing on individual objects, particularly cylinders. Most studies indicate that fluid flow around a single bluff body is mainly influenced by the Reynolds number $(\text{Re}=$ $\frac{{\text{U}}_{\text{in}}\text{d}}{\text{v}};{\text{U}}_{\text{in}}\text{ is inflow velocity},\text{ d is characteristic length and v is kinematic viscosity})$, along with other less influential parameters such as channel blockage ratios (B) and the shape and size of bluff bodies. According to Rajani et al.^1^, fluid flowing around a spherical cylinder exhibits various flow regimes depending on Re, including creeping flow, steady closed near wake, and laminar vortex shedding for Re ranging from 0.1 to 400. Sen et al.^2^ emphasized the onset of flow separation around a stationary circular cylinder within a low Re range, finding that the channel’s blockage ratio influences the onset of flow separations. Zhao^3^ performed numerical analysis of flow around a circular cylinder with a downstream sphere for various Re and gap ratios (G), observing that the shape and size of vortices emerging from the cylinder were greatly influenced by the presence of the sphere in the wake. Saha et al.^4^ evaluated transitions to three-dimensional flow in the wake of a square cylinder for Re from 150 to 500, noting the transition from two- to three-dimensional flow between Re = 150 and 175, and reporting a close correlation between drag force and the Strouhal number $(\text{St}=\frac{{\text{f}}_{\text{s}}\text{d}}{{\text{U}}_{\text{in}}};{\text{ f}}_{\text{s}}\text{ is the shedding frequency})$ within the selected Re range. Zhang et al.^5^ investigated flow around a square cylinder at low Re values of 25, 50, 75, 100, and 150, finding that transitions from symmetrical to asymmetrical flow occur beyond Re = 50. Sen et al.^6^ analyzed fluid flow around a square cylinder at low Re and varying angles of incidence, observing an inverse relationship between Re and wavelength. Islam et al.^7^ examined the influence of a flat plate on flow around a square cylinder for various Re values, reporting four flow patterns: quasi-steady flow (QSF), steady flow (SF), shear layer reattachment (SLR), and single bluff body (SBB) depending on Re and gap ratios. Perumal et al.^8^ conducted lattice Boltzmann computations for two-dimensional incompressible flow past a square cylinder under both steady and unsteady flow regimes, noting the influence of wall boundaries on results, particularly at lower blockage ratios.

Introducing multiple bluff bodies into fluid streams significantly alters fluid flow characteristics, impacting various engineering scenarios. Researchers have explored different arrangements such as inline, side-by-side, and staggered configurations, where the gap distance between bodies and the inclination angle relative to each other play a crucial role in varying flow modes and forces. Rastan et al.^9^ investigated flow and heat transfer across two inline rotating circular cylinders at Re = 100, 150, and 200, with gap ratios of 2.5, 4, and 6, and rotational speeds from -5 to 5, identifying three distinct flow regimes: alternating co-shedding (AC), single rotating bluff body (SRB), and inverted rotation (IR). Mahir et al.^10^ observed double bumps in the local Nusselt number for downstream cylinders at higher Re values in a similar study of flow across two isothermal spherical cylinders in a tandem arrangement. Alam et al.^11^ found that varying diameter ratios (s = d/D) of two circular tandem cylinders resulted in various regimes including lock-in, intermittent lock-in, no lock-in, subharmonic lock-in, and shear layer reattachment. Ahmed et al.^12^ reported that splitter plates attached to tandem square cylinders help control critical flow parameters and flow-induced forces, categorizing flow structures into four regimes: single bluff body (SBB), steady flow (SF), quasi-steady flow (QSF), and fully developed flow (FDF). Sohankar et al.^13^ examined forced convection heat transfer and free-stream flow of power law fluids around single and tandem square cylinders at various Re values, observing stable behavior at Re = 40 and unsteady flow at higher Re values. Yen et al.^14^ studied the effects of Re, G, and rotation angle of the downstream cylinder for flow around two tandem square cylinders, identifying different flow field divisions, including vortex streets of single mode, reattached mode, and binary mode, with variations in St stabilizing after Re = 405. Inoue et al.^15^ explored sound generation mechanisms by flow between two side-by-side square cylinders at low Mach numbers, noting the strong influence of gap ratios on flow fields and sound pressure mechanisms.

A significant arrangement of bluff bodies in civil structures is the staggered arrangement. Previous studies suggest that flows around tandem arrangements are influenced by wake interference, while side-by-side arrangements are affected by proximity effects. In staggered arrangements, flows are influenced by both proximity and wake interference^16^. Nadeem et al.^17^ controlled flow around staggered square cylinders with attached control plates, reporting substantial reductions in drag and lift forces and controlled vortex shedding. Chauhan et al.^18^ found that fluid flow past staggered cylinders depends on G and inclination angle. Fezai et al.^19^ investigated the impact of staggered arrangements on incompressible fluid flow, noting marginal effects on drag and lift force fluctuations. Islam et al.^20^ observed complex flow structures at narrow gaps and stable periodic flow at higher gaps in staggered square cylinder arrangements across various Re values. Bhatt et al.^21^ found that the position and height of upstream cylinders affect wake flow patterns in staggered arrangements. Niu et al.^22^ noted correlated drag and lift forces between upstream and downstream cylinders in staggered arrangements. Manshoor et al.^23^ observed increased sound levels near cylinder surfaces at higher Re values when detached flat plates influenced sound generation and vorticity in staggered square cylinder flow. Aboueian et al.^24^ reported that flow patterns typically seen in side-by-side arrangements also emerge in staggered arrangements.

Recent literature has continued to advance the understanding of fluid dynamics around bluff bodies, particularly in staggered arrangements. For instance, Smith et al.^35^ conducted an in-depth analysis of fluid flow around staggered cylindrical arrays using high-fidelity simulations, highlighting the impact of gap ratios on vortex shedding and pressure distributions. Their findings emphasize the complex interactions between proximity effects and wake interference, which corroborate and extend our investigation into the transitional flow regimes. Similarly, Chen and Wang^36^ explored the effect of varying Reynolds numbers on the stability of flow patterns in staggered cylinder configurations, providing new insights into the conditions that lead to synchronized and flip-flopping flow states. These studies underscore the necessity of considering a wider range of flow parameters to fully capture the dynamics in staggered arrangements. Additionally, recent advancements in computational methods have furthered the precision of simulations in fluid dynamics research. Johnson et al.^37^ applied the Lattice Boltzmann Method (LBM) to study the fluid–structure interactions in staggered bluff body configurations, demonstrating significant improvements in computational efficiency and accuracy. Their work supports our methodological choice and highlights the versatility of LBM in complex flow scenarios. Furthermore, Zhang et al.^38^ investigated the aerodynamic forces on staggered rectangular cylinders at various angles of attack, revealing critical dependencies of drag and lift forces on both geometric and flow parameters. These contemporary studies provide a robust foundation for our research, enabling us to refine our analysis and contribute novel insights into the fluid dynamics of staggered cylinder arrangements.

From the above-discussed literature, it is evident that the arrangement of multiple bluff bodies significantly influences the flow structures and force variations around these bodies. Despite previous investigations into the flow characteristics of staggered cylinders^16–24^, this area remains underexplored and warrants further attention. Staggered arrangements, frequently seen in many engineering applications, demand thorough and detailed investigations. These arrangements are influenced by the combined effects of proximity and wake interference, and the available information is far from complete. To address these shortcomings and unresolved issues, this paper aims to study the flow features of two square cylinders in a staggered arrangement. Using the Lattice Boltzmann Method (LBM), our objective is to analyze the impact of cylinder spacing on flow structure mechanisms and the variation of fluid-induced forces at different cylinder positions.

The key fluid flow parameters under investigation include vorticity behavior, streamline patterns, variations in drag coefficient $(\text{CD}=\frac{2{\text{F}}_{\text{d}}}{\uprho {\text{U}}_{\infty }^{2}\text{d}}; {\text{F}}_{\text{d}}\text{ is the drag force})$ and lift $\left(\text{CL}=\frac{2{\text{F}}_{\text{l}}}{\uprho {\text{U}}_{\infty }^{2}\text{d}};{\text{F}}_{\text{l}}\text{ is the drag force}\right)$ coefficients, root mean square (rms) values of drag and lift coefficients, and variations in Strouhal number. This study seeks to provide insights into the complex fluid dynamics of staggered cylinder arrangements, contributing to a more comprehensive understanding of multi-body flow interactions.

### Our scientific problem and gap in prior researches

The scientific problem this study aims to address is the incomplete understanding of fluid flow characteristics and force variations around staggered arrangements of square cylinders. Despite previous investigations into the flow characteristics of staggered cylinders, this area remains underexplored. Prior research has focused predominantly on single bluff bodies or other configurations like tandem and side-by-side arrangements, leaving a significant gap in the comprehensive analysis of staggered arrangements. The combined effects of proximity and wake interference in staggered configurations are not thoroughly understood, particularly their influence on flow structures, pressure variations, and fluid-induced forces.

### Aims of the study

The primary aims of this study are:

1. To investigate the fluid flow characteristics around two staggered square cylinders using the Lattice Boltzmann Method (LBM).
2. To analyze the impact of varying gap ratios (G) on flow structure mechanisms and induced forces at different cylinder positions.
3. To identify and characterize distinct flow patterns emerging from different gap ratios.

### Novelty

The novelty of this study lies in its detailed computational analysis of fluid flow around staggered square cylinders, an area with limited prior research. Key novel contributions include:

1. A comprehensive exploration of flow structure mechanisms and induced forces at varying gap ratios, providing new insights into the behavior of fluid flow around staggered cylinders.
2. Identification and characterization of four distinct flow patterns (single bluff body flow, flip flopping flow, modulated synchronized flow, and synchronized flow) based on the gap ratio, which has not been extensively reported in previous studies.
3. Use of the Lattice Boltzmann Method (LBM) to analyze the complex interactions in staggered cylinder arrangements, contributing to a deeper understanding of how cylinder spacing influences vorticity behavior, pressure streamlines, drag, and lift coefficients, as well as the Strouhal number.

#### Lattice Boltzmann method

In this study, we will utilize the Lattice Boltzmann method for numerical computations, which was developed in 1988^25^. This method is applicable for solving fluid dynamics problems, multiphase flows, solidification and melting problems, among others. LBM utilizes both macroscopic and microscopic levels and is easily applicable in complex domains with intricate boundaries. The Boltzmann equation is

1 $$\frac{\partial \text{g}}{\partial \text{t}}+\text{c}.\nabla \text{g}=\Omega$$

Therefore, c and $\nabla \text{g}$ are vectors quantities.

Equation (1) is advection equation. This equation relates with macroscopic quantities as.

#### Density

2 $$\uprho (\text{r},\text{t})=\int \text{mg}(\text{r},\text{c},\text{t})\text{dc}$$

#### Velocity

3 $$\uprho (\text{r},\text{t})\text{u}(\text{r},\text{t})=\int \text{mcg}(\text{r},\text{c},\text{t})\text{dc}$$

It is very hard to solve a Boltzmann equation due to its collision term. It is possible to approximate the collision operator with simple operator without introducing some error to the solution. In 1954, Bhatnagar Gross and Krook (BGK) introduced a simplified model for collision operator^26^.

4 $$\Omega =\upomega \left({\text{g}}^{\text{eq}}-\text{g}\right)=\frac{1}{\uptau }\left({\text{g}}^{\text{eq}}-\text{g}\right)$$

where $\uptau$ shows relaxation parameter and ${\text{g}}^{\text{eq}}$ is local equilibrium distribution function.

After BGK approximation the Boltzmann equation becomes

5 $$\frac{\partial \text{g}}{\partial \text{t}}+\text{c}.\nabla \text{g}=\frac{1}{\uptau }\left({\text{g}}^{\text{eq}}-\text{g}\right)$$

Discretizing the above equation, we get

6 $$\frac{\partial {\text{g}}_{\text{i}}}{\partial \text{t}}+{\text{c}}_{\text{i}}\nabla {\text{g}}_{\text{i}}=\frac{1}{\uptau }({\text{g}}_{\text{i}}^{\text{eq}}-{\text{g}}_{\text{i}})$$

The above equation is liner PDE. It looks like an advection equation with a source term. Discretizing the above equation, we get

7 $${\text{g}}_{\text{i}}(\text{r}+{\text{c}}_{\text{i}}\Delta \text{t},\text{t}+\Delta \text{t})={\text{g}}_{\text{i}}(\text{r},\text{t})+\frac{\Delta \text{t}}{\uptau }[{\text{g}}^{\text{eq}}(\text{r},\text{t})-\text{g}(\text{r},\text{t})]$$

The above equation is the main working equation in LBM simulations. It can recover the so-called Navier–Stokes equations through the Chapmann-Enskog expansion^26^. Note that, in the current study we have considered a uniform meshing in the whole computational domain. The spatial as well as temporal step size is taken to be 1 i.e $\Delta \text{x}=\Delta \text{t}=1$. The equilibrium distribution function involved in above equation is

8 $${\text{g}}_{\text{i}}^{\text{eq}}=\upphi {\upomega }_{\text{i}}\left[\text{A}+{\text{Bc}}_{\text{i}}.\text{u}+\text{C}{\left({\text{c}}_{\text{i}}.\text{u}\right)}^{2}+{\text{Du}}^{2}\right]$$

The density and momentum in LBM are computed by the relations^25^:

#### Density

9 $$\uprho ={\sum }_{\text{i}=0}^{\text{M}-1}{\text{g}}_{\text{i}}={\sum }_{\text{i}=0}^{\text{M}-1}{\text{g}}_{\text{i}}^{\text{eq}}$$

#### Momentum

10 $$\uprho {\text{u}} = \sum\limits_{{\text{i}} = 0}^{{\text{M}} - 1} {{{\text{c}}_{\text{i}}}{{\text{g}}_{\text{i}}}} = \sum\limits_{{\text{i}} = 0}^{{\text{M}} - 1} {{{\text{c}}_{\text{i}}}{\text{g}}_{\text{i}}^{{\text{eq}}}}$$

**Figure 1:**
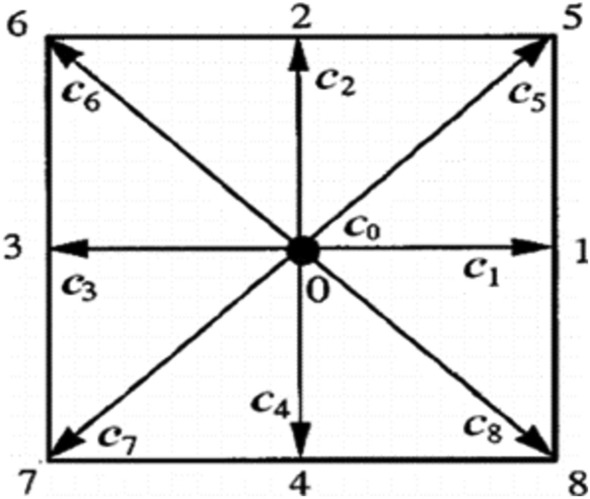
D2Q9 particles distribution.

In this study, we will consider two-dimensional and nine velocity-particle model i.e. D2Q9 model. It is very common for solving fluid flow problems (Fig. 1). The weighting factors of distributions functions are

$$\omega_{0} = {\raise0.7ex\hbox{$4$} \!\mathord{\left/ {\vphantom {4 9}}\right.\kern-0pt} \!\lower0.7ex\hbox{$9$}},\,\omega_{a} = {\raise0.7ex\hbox{$1$} \!\mathord{\left/ {\vphantom {1 9}}\right.\kern-0pt} \!\lower0.7ex\hbox{$9$}},\,\omega_{d} = {\raise0.7ex\hbox{$1$} \!\mathord{\left/ {\vphantom {1 {36}}}\right.\kern-0pt} \!\lower0.7ex\hbox{${36}$}}.$$

where ${\upomega }_{0}$ for the rest particle, ${\upomega }_{\text{a}}$ for particles moving along axis and ${\upomega }_{\text{d}}$ for particles moving along diagonal directions.

Further details and applications of the Lattice Boltzmann equation can be found in Mohamad^27^.

## Geometry of the problem

**Figure 2:**
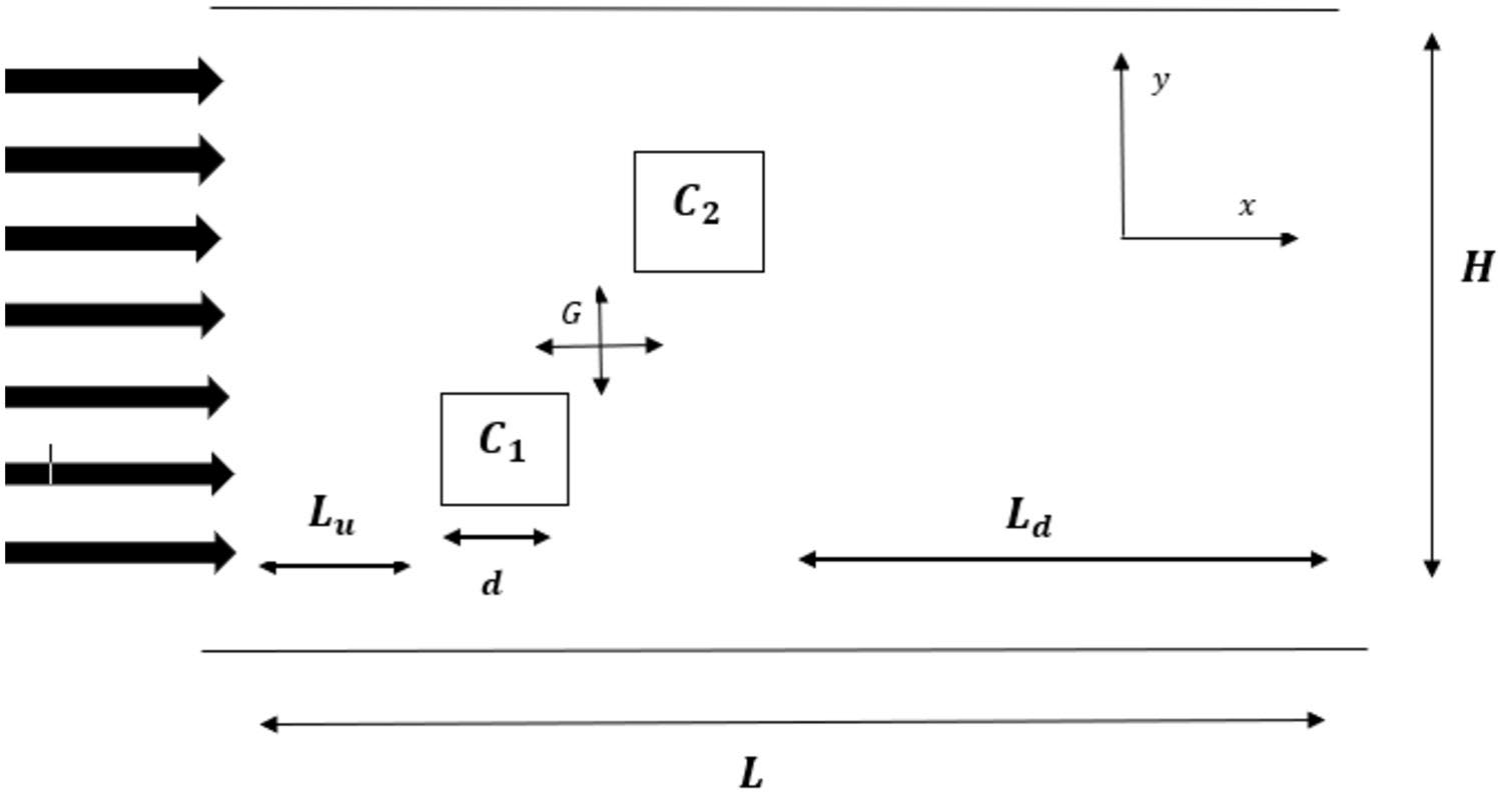
Schematic diagram for flow around two staggered arranged square cylinders.

The schematic flow diagram of the proposed problem of flow around two staggered square cylinders is presented in Fig. 2. In this figure, d represents size of the obstacles, ${\text{L}}_{\text{u}}$ is upstream length from inlet to the first cylinder (C_1_) and ${\text{L}}_{\text{d}}$ is downstream length from second cylinder (C_2_) to outlet position of the channel. L is the length and H is the height of domain while G is gap ratio between the cylinders. For current analysis, we have taken $\text{d}$ = 20, ${\text{L}}_{\text{u}}$= 8d, ${\text{L}}_{\text{d}}$ = 16d. The values of L and H depend on G which varies from 0 to 10 times of size of the cylinder. The boundary conditions considered in this study are given in Table 1. This table indicates that the fluid enters the domain with uniform velocity $(\text{u}={\text{U}}_{\text{in}}:\text{v}=0)$. The solid walls of channel and the cylinders are modeled through the no-slip condition $(\text{u}=\text{v}=0)$ using the bounce back rule^27^. At exit position, to conserve the mass and momentum of system the convective boundary condition ${\partial }_{\text{t}}\text{g}+{\text{U}}_{\text{in}}{\partial }_{\text{u}}\text{g}=0$ is applied^17^.

**Table 1:** Boundary conditions at different positions for flow past two staggered arranged square cylinders.

Location	Inlet	Outlet	Solid walls
Boundary conditions	$\text{u}={\text{U}}_{\text{in}}$ $\text{v}=0$	${\partial }_{\text{t}}\text{g}+{\text{U}}_{\text{in}}{\partial }_{\text{u}}\text{g}=0$	$\text{u}=\text{v}=0$ (No slip condition)

## Grid independence and code validation

### Grid Independence

In order to get results independent of grid size, we have performed grid independence study at Re = 200 by taking 10-points, 20-points and 40-points grid lattices at surface of the cylinder (Table.2). Note that 10-points grid means that each surface of cylinder (left, right, upper, and lower) is subdivided in 10 lattices. Similar is the case for 20- and 40-points grid. In Table 2, the 0.8%, 2%, 0.9% etc. are the percentage differences of the Cdmean, St and Clrms values corresponding to 10- and 20-points grids from the 40-points grid size. It is apparent from Table 2. that 20-points grid gives fair results regarding computational time and accuracy. So, we will consider 20-points gird for analysis in this research. The 20-points lattices were also used by Islam et al.^33^. According to them it is perfect grid size which ensures accuracy at low computations cost.

**Table 2:** Effect of spatial resolution on fluid flow parameters for flow past a single square cylinder at Re = 200.

	10-points	20-points	40-points
Cd-mean	1.5214 (0.8%)	1.5198 (0.7%)	1.5086
St	0.1518 (2%)	0.1549 (0%)	0.1549
Cl-rms	0.4496 (0.9%)	0.4534 (0.09%)	0.4538

### Code validation

To verify our code quantitatively, we have computed average drag coefficient, Strouhal number and root mean square value of drag and lift coefficient for flow past a square cylinder at Re = 200 and compared our present results with other researchers as shown in Table 3. Our results agree well with those of other researchers with minor deviations. This indicates the authenticity of our code for such type of problems.

**Table 3:** Comparison of Cd-mean, St, Cd-rms and Cl-rms for flow past a single square cylinder at Re = 200.

	Saha et al.^28^	Sohankar et al.^29^	Okajima^30^	Norberg^31^	Abograis and Aishayji^32^	Present
Cd-mean	1.670	1.424	1.480	1.450	1.488	1.519
St	0.163	0.165	0.138	0.152	0.153	0.155
Cd-rms	–	0.012	–	–	0.027	0.038
Cl-rms	–	0.240	–	–	0.332	0.453

**Figure 3:**
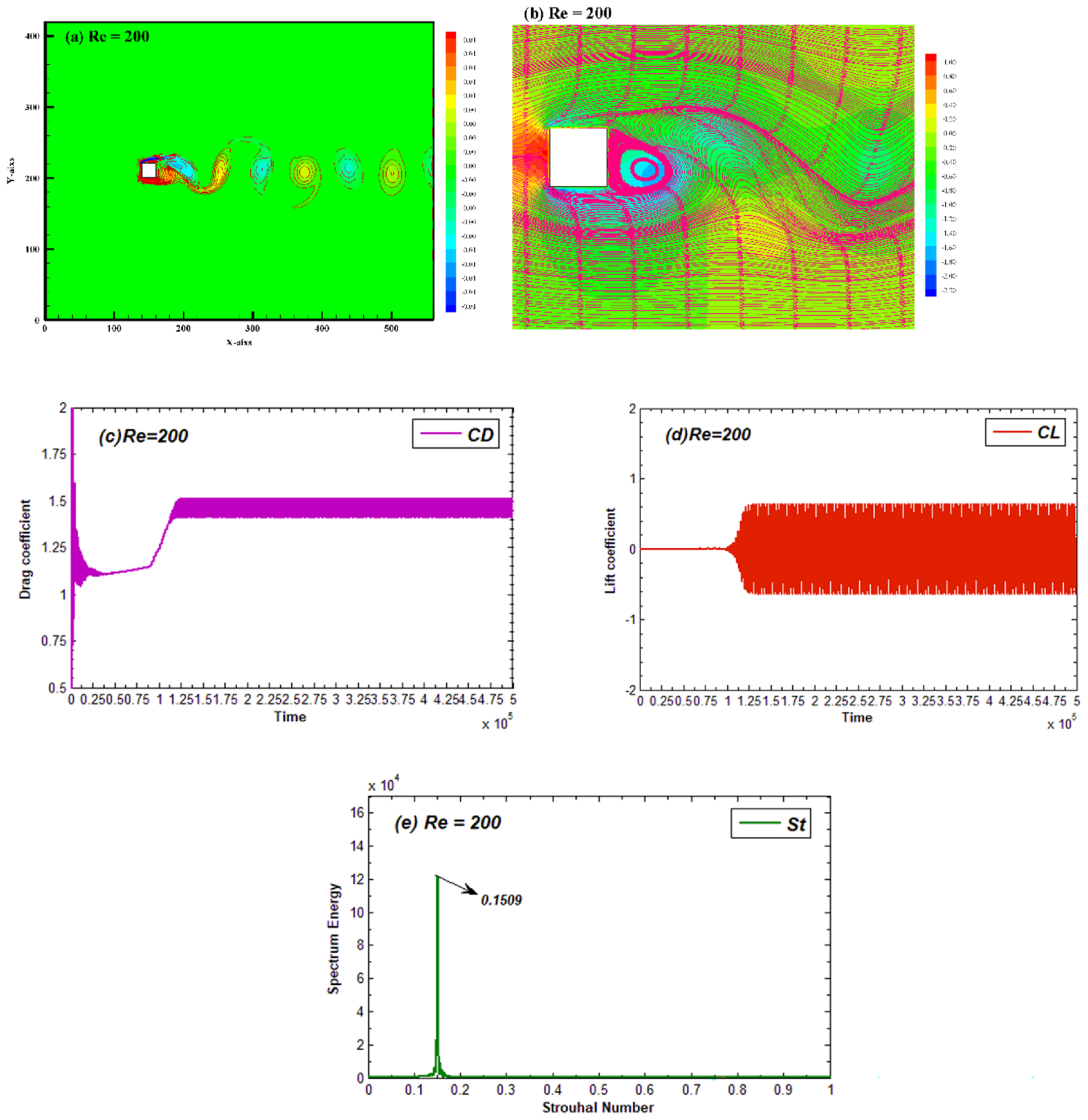
(**a**) Vorticity contour, (**b**) Pressure streamlines (**c**,**d**) Time-histories of CD and CL, and (**e**) power spectrum of CL for flow around single square cylinder at Re = 200.

Before analyzing the flow past two staggered arranged square cylinders, we have studied the flow past a single square cylinder at Re = 200 presented in Fig. 3. Figure 3a represents the vorticity contour for the flow around a square cylinder at Re = 200. It can be observed that the fluid after splitting from the cylinder roll up in wake and form the vortices represented as the rotating fluid either in clockwise or anticlockwise direction termed as negative vortices (represented with dashed lines) and positive vortices (represented with solid lines), respectively. The streamlines indicating the direction of fluid movement are shown in Fig. 3b. Waviness of streamlines indicates the unsteady behavior of flow due to generation of vortices in the wake. The recirculation zone can be observed adjacent to the cylinder’s back surface which shows the vortex formation region. Whenever the fluid interacts to an object, it exerts forces on the object. Similar is the current case in which the fluid interacting with cylinder exerts the drag and lift forces on cylinder. Time history dynamics of both the force’s coefficients, that is, CD and CL are plotted at different time steps in Fig. 3. It can be seen from Fig. 3c,d that initially the CD signals appear randomly but after some time steps the CD settles down to a repeated periodic like behavior. Contrary to that, the CL attains such variations after being steady for a short interval of time. Figure 3e shows the power spectrum of lift coefficient in which a single peak appears indicating the smooth generation of vortices from cylinder. The single peak in power spectrum of CL refers to no modulation in lift coefficient and periodicity of CL.

Such fluid flow characteristics around a single square cylinder have been frequently reported in literature. The purpose of reproduction of such results is to authenticate the code used for current analysis. From the above discussion it is apparent that the code used for current study is capable for computing the fluid flows around bluff bodies.

## Results and discussion

In this work, after the validation study the influence of G on the flow past two staggered arranged square cylinders is examined. We have taken different gap ratios and Reynolds number is fixed at Re = 200. The computational results are analyzed in terms of flow behavior from vorticity contour, pressure streamlines, time dependent variation of drag and lift coefficient and power spectrum analysis of CL.

In our study, the gap ratios considered range from 0 to 10 times the size of the cylinder (0 ≤ G ≤ 10). This parameter is crucial in determining the interaction between the flow fields around the cylinders and influences the resulting flow patterns, vortex structures, and force coefficients. Now we will discuss various flow patterns observed for the flow around two staggered arranged square cylinders at different G and Re = 200. This numerical study reveals four different flow patterns corresponding to different values of G named as the single bluff body (SBB) flow at G = 0, flip flopping (FF) flow in the range 0.5 ≤ G ≤ 2.5, modulated synchronized (MS) flow in the range 3 ≤ G ≤ 5 and synchronized flow (SF) in the range 6 ≤ G ≤ 10. The following subsections present the detailed characteristics of each of these flow patterns:

### Single bluff body flow

**Figure 4:**
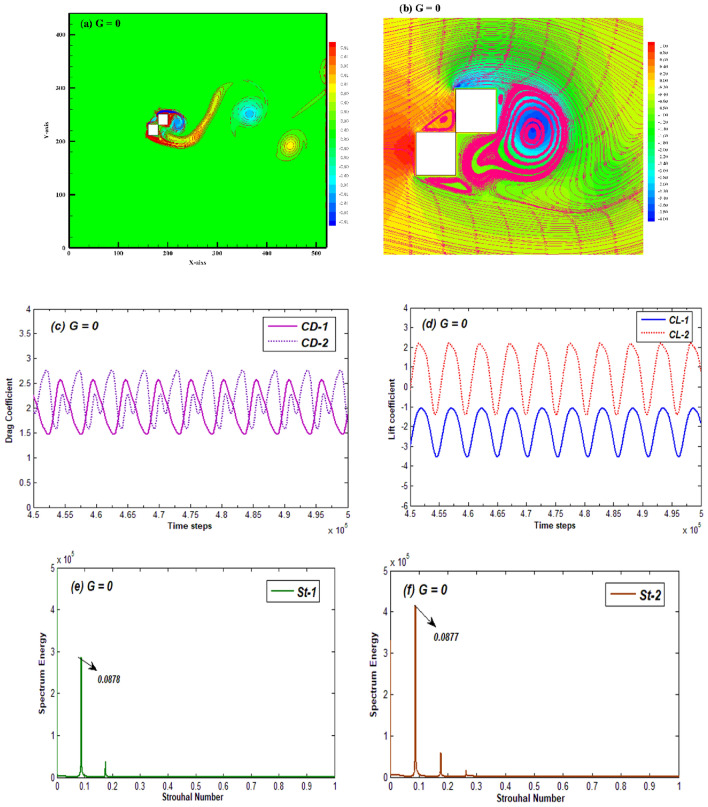
(**a**) Vorticity contour, (**b**) Pressure streamlines (**c**,**d**) Time- histories of CD and CL, and (**e**,**f**) power spectrum of CL for the Single Bluff Body flow pattern at G = 0 and Re = 200.

As revealed by previous studies, when gap ratio is sufficiently small (negligible) between the cylinders, multiple bluff bodies look like a single body and the associated flow behavior termed as single bluff body flow^24^. Similar is the present case in which the SBB flow is observed at G = 0. Its prominent characteristics are described through vorticity contour, pressure streamlines, time dependent variations of CD and CL, and power spectrum of CL (Fig. 4). The vorticity contour shows that the flow separation is restricted to the upper corner of ${\text{C}}_{2}$ and the lower corner of ${\text{C}}_{1}$ without any gap flow due to no spacing between cylinders (Fig. 4a). Both negative and positive vortices combine and form a single wake in which the vortices apparently do not have a definite shape. As can be seen, an elongated vortex structure appears initially emerging from lower side of first cylinder, which is then followed by an elliptic vortex structure. Islam et al.^33^ also reported such flow pattern in their study for flow around staggered cylinders at low spacing value. Figure 4b represents pressure streamlines behavior corresponding to SBB flow. It can be observed that the pressure is maximum at front face of ${\text{C}}_{1}$ and minimum at upper corner of ${\text{C}}_{2}$. Multiple eddies appear at different positions adjacent to cylinders indicating the recirculation zones. Such zones emerge due to shear layers’ detachment from cylinders’ sides and merging in the wake. The large recirculating eddy appearing in the wake of both cylinders indicates the point where shear layers merge and thus generate vortices. The time dependent variation of $\text{CD}$ and $\text{CL}$ of both cylinders corresponding to SBB flow exhibits the periodic behavior with slight modifications in amplitude of consecutive cycles for both ${\text{C}}_{1}$ and ${\text{C}}_{2}$ (Fig. 4c,d). This periodicity of CD and CL results from smooth detachment of shear layers from cylinder’s corners and thus shedding regular vortices in the wake. Due to periodicity in CL, the power spectrum graph displays a single leading peak together with small secondary peaks for both the cylinders (Fig. 4e,f). These small peaks appear due to the modulated vortex structures in the wake of cylinders. Figure 4e,f also shows that the value of Strouhal number for first cylinder (St-1) is almost like that for the second cylinder. This indicates that the vortices from both cylinders shed at same frequency.

### Flip flopping flow

**Figure 5:**
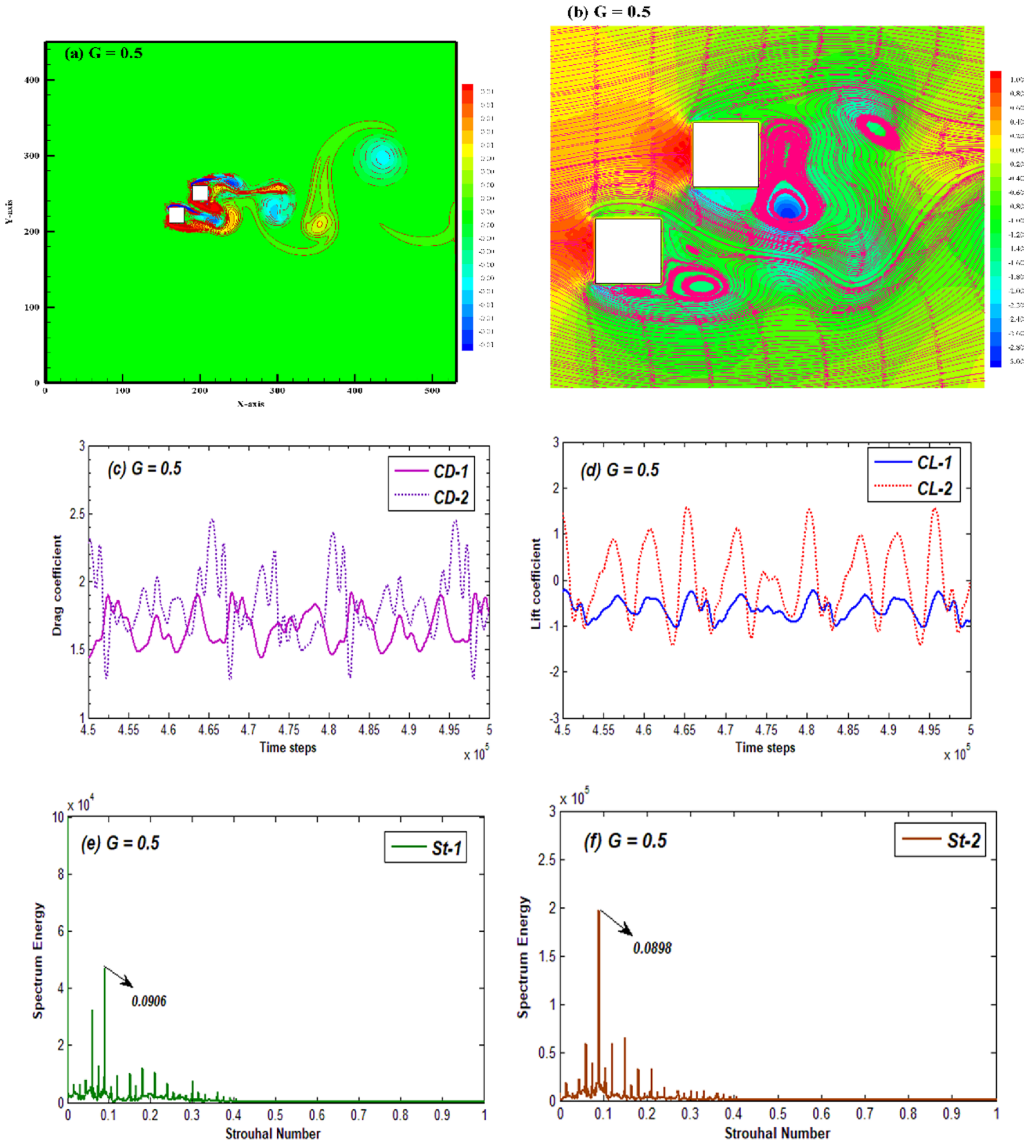
(**a**) Vorticity contour, (**b**) Pressure streamlines (**c**,**d**) Time-histories of CD and CL, and (**e**,**f**) power spectrum of CL for the flip flopping flow pattern at G = 0.5 and Re = 200.

When the G between cylinders is successively increased, the flow pattern changes from SBB to flip flopping (FF) flow. This flow pattern spans over the spacing range G = 0.5–2.5. A representative case for FF flow at G = 0.5 is presented in Fig. 5. In this flow pattern, the wake structure is significantly disrupted due to strong effect of jet flow emerging from the gaps between cylinders (Fig. 5a). The vortices in the wake of both cylinders appear to be non-regular owing to the amalgamated structures leaning towards upper side of the computational domain. From previous studies it can be inferred that in the flip flopping pattern the jet flow significantly disturbs the wake dynamics^17^. It normally emerges in the narrow gaps between the bodies. Due to flip flopping nature of flow, the pressure switches randomly at different parts inside the channel. Like the SBB flow, the pressure is maximum at front sides of both cylinders but in this case the low-pressure zone shifted towards the lower front corners of both cylinders and in the region where jet flow emerges unlike the SBB flow (Fig. 5b). The complex flow structures in the wake region are also apparent from streamlines behavior in Fig. 5b. The eddies adjacent to cylinders as well as in the wake appear inconsistently having diverse structures. This complexity in flow structure also affects the flow induced drag and lift forces (Fig. 5c,d). Both force coefficients randomly switch between higher and lower values and the periodicity that was observed in case of SBB flow is no longer perceptible. Figure 5c,d shows that the drag coefficients on both cylinders vary in an antiphase fashion while lift coefficient vary in-phase to each other. It is important to mention here that the jet flow effect exploits till G = 1 and it gradually decreases with further increase in spacing between the cylinders. This results in periodicity of lift coefficient of first cylinder, but the drag remains modulated (Figures not shown for brevity). Due to random fluctuations in the lift coefficient, multiple peaks appear in the power spectrum of CL for both cylinders (Fig. 5e,f). Unlike the SBB flow pattern the St for both cylinders differ considerably from each other due to the substantial differences in shedding frequencies from both cylinders. Note that in this flow pattern, as the gap ratios increase, and lift stabilizes the multiple peaks no longer appear in power spectrum of CL. Chatterjee et al.^34^ also reported flip flopping pattern for smaller gap ratio (G = 1). Similar characteristics of FF flow pattern were described by Refs.^15^^,^^17^.

### Modulated synchronized flow

**Figure 6:**
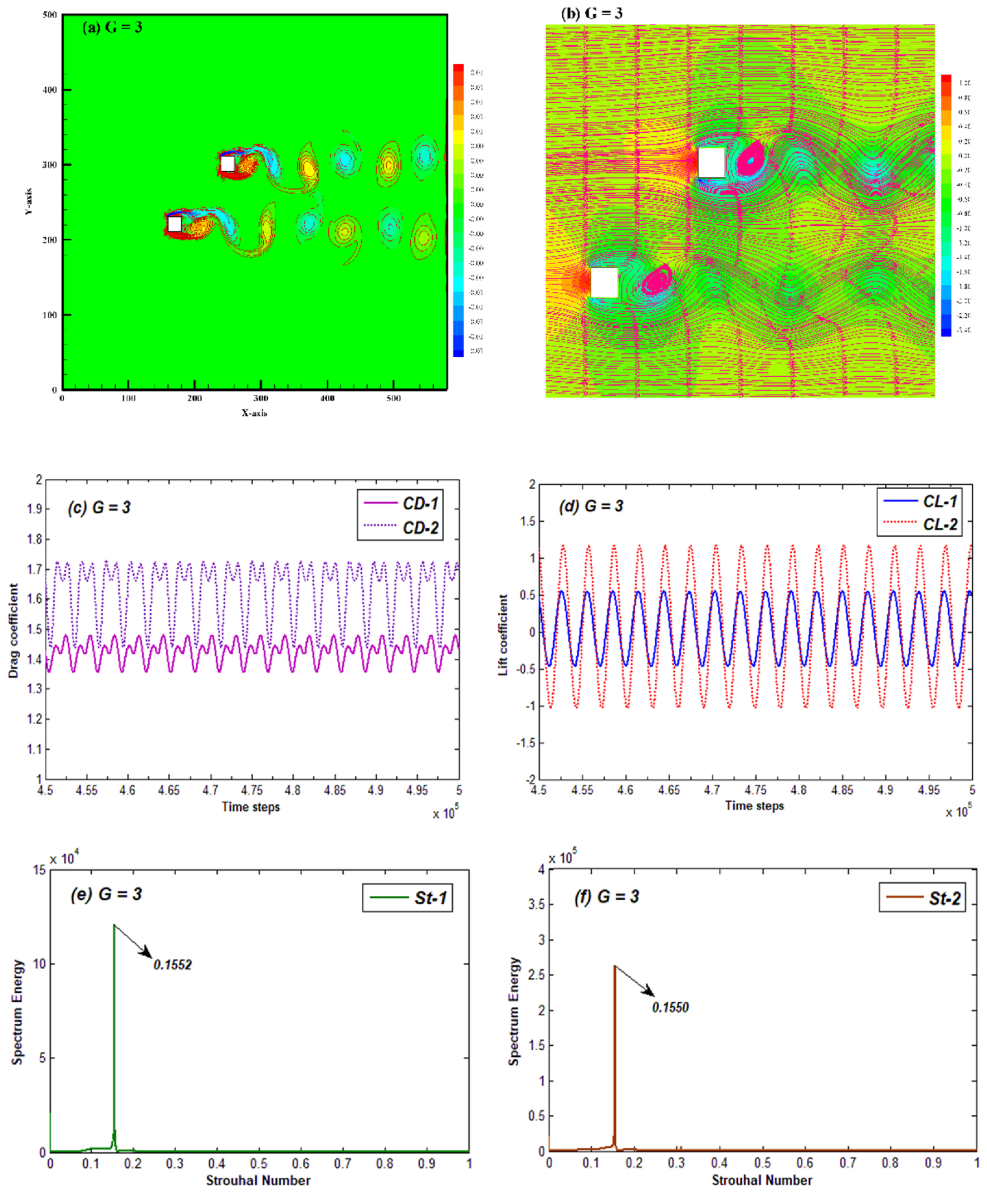
(**a**) Vorticity contour, (**b**) Pressure streamlines (**c**,**d**) Time-histories of CD and CL, and (**e**,**f**) power spectrum of CL for the Modulated synchronized flow pattern at G = 3 and Re = 200.

As described earlier, the impact of jet flow between the gaps minimizes as the distance between cylinders increases. In such cases, the proximity effects become weaker, and a new flow pattern appears which is termed as the modulated synchronized (MS) flow. The MS flow is observed in this study at moderate gap ratios G = 3–5. For discussion purposes, we have considered only G = 3 as a representative case. The resulting vorticity contour, pressure and streamlines variations, temporal variation of lift and drag coefficient and power spectrum of lift coefficient for this case are shown in Fig. 6. Figure 6a shows that a separate vortex street emerges from each cylinder in which vortices travel alternately in a synchronized pattern without merging with other cylinder’s wake. The jet flow effect seems to have vanished due to negligible proximity of cylinders. Shedding pattern switches between the in-phase and anti-phase developed vortices from both cylinders as the spacing between cylinders changes. The streamlines variation also outlines the separate vortex street pattern in wake of each cylinder (Fig. 6b). The solo eddy developed in the vicinity of each cylinder points the shear layers merging region. This figure also depicts the appearance of separate low-pressure zones in the wake of each cylinder with modulated pressure zones in the gap region area. This synchronized flow, over a period, leads to an amplitude modulation in drag coefficient of both cylinders while the lift appears to be fully periodic due to separate vortex streets (Fig. 6c,d). Due to synchronized wakes and modulated drag force this flow pattern is categorized as MS flow. The drag as well as lift force on second cylinder have higher amplitude as compared to first cylinder. This indicates that the proximity effects of each cylinder on other’s wake dynamics are weakening, and each cylinders’ wake behaves distinctly. Also, both force coefficients vary in an in-phase fashion due to initial same sign vortex development. The power spectrum graph for MS flow shows the single dominant peak. Note that like the SBB flow pattern both St-1 and St-2 have approximately the identical values indicating the similarity in shedding frequencies from both the cylinders (Fig. 6e,f). This type of flow pattern was named as modulated periodic flow by Aboueian and Sohankar^24^.

### Synchronized Flow

**Figure 7:**
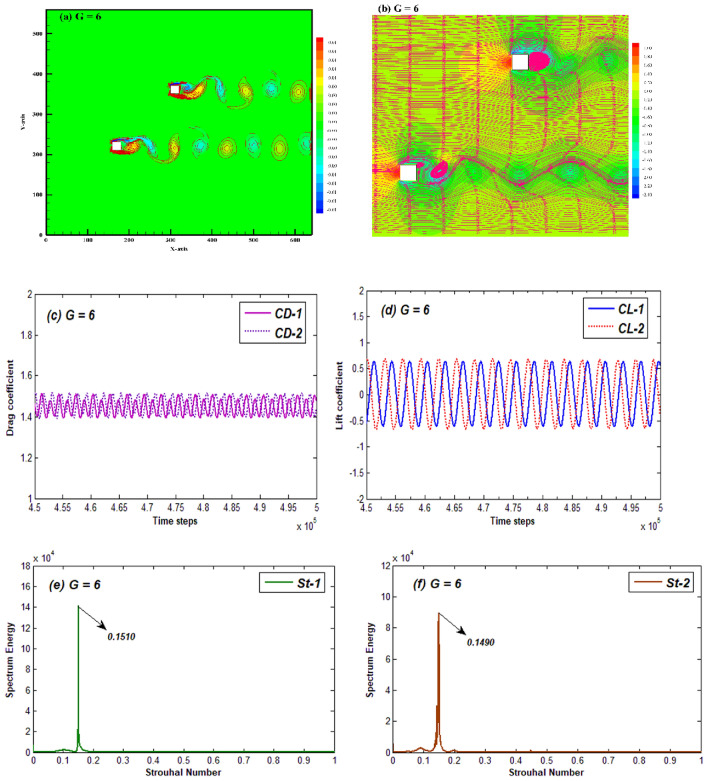
(**a**) Vorticity contour, (**b**) Pressure streamlines (**c**,**d**) Time-histories of CD and CL, and (**e**,**f**) power spectrum of CL for the synchronized Flow pattern at G = 6 and Re = 200.

When the gap ratio between the cylinders is sufficient, that is, in the range G = 6–10 the flow pattern changes its behavior from MS flow to synchronized flow (SF). The representative case of this flow pattern is given for G = 6, the first gap ratio owing this flow pattern (Fig. 7). In this case, the influence of each cylinder on other’s wake flow characteristics vanishes and the flow features around each cylinder are approximately like those for a single square cylinder. As it can be seen from vorticity contour representing the SF case that each cylinder’s wake resembles to the solo cylinders’ Karman vortex street (Fig. 7a). The streamlines contour also shows that the effect of gap flow observed at narrow spacings between cylinders is no longer prevalent (Fig. 7b). Each cylinder has a separate flow field and produces an oscillating wake in the form of two individual streamlined rows. Three recirculating zones appear in this flow pattern: a larger eddy seems to be attached with ${\text{C}}_{2}$ while two small eddies can be observed in the wake of ${\text{C}}_{1}$, one is attached with the upper corner of ${\text{C}}_{1}$ and other is slightly away from the cylinder. Such smaller recirculating eddies indicate smaller recirculating regions for vortices resulting in smaller size vortex structures as compared to other flow patterns. Time history analysis of force coefficients indicates that both CD and CL vary in a periodic fashion (Fig. 7c,d). The amplitude of both these forces is less as compared to other flow patterns which is due to smaller size vortices appearing in this case. Due to the periodicity in CL and smooth passage of vortices the power spectrum shows a single dominant peak for both cylinders (Fig. 7e,f).

## Force statistics

**Figure 8:**
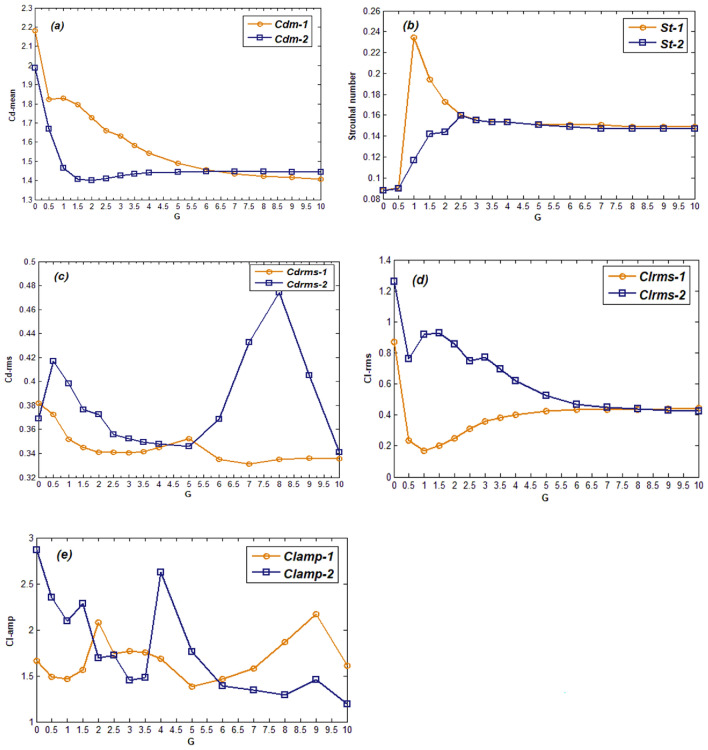
Variation of (**a**) Mean of drag coefficient (**b**) Strouhal number (**c**,**d**) Root mean square of drag and lift coefficient (**e**) Amplitude of lift coefficient with gap spacing.

In the case of the fluid flows around bluff bodies, the altering flow structures have a direct impact upon the flow parameters. The important flow parameters include the mean drag coefficient (Cd-mean), Strouhal number, root mean square values of drag and lift coefficient (Cd-rms) and (Cl-rms) and the amplitude of lift coefficient (Cl-amp). The variation of such force parameters for both cylinders, under the effect of changing G and hence the flow patterns, is discussed in this section. Figure 8a indicates that the Cd-mean of first cylinder (Cdm-1) decreases gradually with increasing G. While that of second cylinder decreases initially till the point where flip flopping flow pattern changes to the MS flow at G = 2.5. This might be attributed to the weakening effect of jet flow on the first cylinder which results in decrease of drag force on first cylinder. While the second cylinder bears little influence of jet flow till the last spacing value being in the wake of first cylinder. In the case of SBB, FF and MS patterns, that is, 0 ≤ G ≤ 5 the Cdm-1 is greater than Cdm-2. Both Cdm-1 and Cdm-2 have the local maxima at G = 0 for SBB flow due to enlarged body size as there is no gap. In Fig. 8b the variation of St of both cylinders is shown which is computed by applying Fast Fourier Transform (FFT) on lift coefficient. This figure indicates that both the St-1 and St-2 differ from each other for gap spacing values between 0.5 to 2.5. This spacing range owns the FF pattern in which the flow structure in the wake of both cylinders differs significantly. At all other spacing values since a regular vortex shedding was observed that’s why the St is almost similar for both cylinders. Note that the time history analysis of CL also indicates irregular variations in CL for such spacing values. Contrary to the St, the Cdrms for both cylinders do not follow regular variation in values, instead vary randomly as described from Fig. 8c. These random fluctuations indicate that the drag coefficients differ from the mean values irrespective of the gap spacing. This is mainly due to variations in the consecutive cycles as well as the amplitudes of drag coefficients for all flow patterns. The Cdrms-2 shows two peaks, the first one at G = 0.5 due to the strong effect of jet flow between the cylinders and due to the flow pattern change from SBB to FF. The second peak of Cdrms-2 occurs at G = 8 in the case of synchronized flow pattern where both cylinders flow characteristics resemble the single square cylinder. It is evident from Fig. 8d that unlike CD-rms, the Cl-rms values of first cylinder either increase or decrease with successive increase in the gap ratios while the Cl-rms of second cylinder fluctuate randomly. Initially Clrms-1 is lower than Clrms-2 but for large G both become almost same due to synchronization of flow which results in a regular lift signal. The effect of increasing G on the amplitude of lift coefficient of both cylinders is shown in Fig. 8e. This figure is evident that the detaching flow from cylinder as well as the formation mechanism of flow structures directly affects the lift force amplitude. The maximum amplitude of CL for first cylinder can be seen at G = 9 in case of the SF pattern while it is minimum at G = 5 in case of the MS flow pattern. On the other hand, the maximum value of Clamp-2 occurring at G = 0 indicates that for SBB flow the amplitude of fluctuating lift is maximized for this case. Note that at G = 0 the vortex formation region was increased due to enlarged cylinders size and it deflected towards the second cylinder which resulted in the higher amplitude lift cycles. While the minimum Clamp-2 at G = 10 is due to the smaller vortex formation regions in the SF pattern case.

## Conclusion

This numerical study analyzed the fluid flow characteristics around two staggered arranged square cylinders. The influence of cylinders’ spacing on flow structure and the flow induced forces was analyzed in detail. The cylinders’ spacing were considered in the range of G = 0 to 10 with a fixed Reynolds number (Re = 200) and a fixed angle of inclination of 45° between cylinders. The results were discussed in terms of vortex generation mechanism, pressure streamlines, time history analysis of lift and drag coefficients, and the power spectrum of the lift coefficient. A summary of the important findings of this analysis is provided below:

1. This numerical study reveals that the gradual variation in gap spacing between cylinders results in alteration of flow patterns. Based on such alterations four different flow patterns were identified: the single bluff body flow, flip flopping flow, modulated synchronized flow, and synchronized flow.
2. The single bluff body flow was found at G = 0 only, where both cylinders act like a single body in flow field. In this flow pattern, the elongated vortex structures appeared in the wake eventually deflecting to the upward region of domain. Both cylinders faced higher amplitude drag and lift forces in this flow pattern.
3. Flip flopping flow was observed for 0.5 ≤ G ≤ 2.5. In this flow pattern, the vortices in the wake of both cylinders appeared to be chaotic due to the strong influence of the jet flow. The drag as well as lift force coefficients randomly fluctuated with high modulations and varying amplitudes of succeeding cycles. The shedding frequencies of vortices were also higher in this flow pattern as compared to other flow patterns case.
4. In the range of 3 ≤ G ≤ 5, modulated synchronized flow was observed. In this flow pattern jet flow effect gradually minimized and synchronized vortices generated in the wake of both cylinders. The random fluctuations in the fluid forces settled down as spacing progressively increased. The modulations in the amplitudes of CD for both cylinders occurred while CL appeared to be wholly periodic.
5. Synchronized flow pattern was observed in the range of 6 ≤ G ≤ 10 without any significant proximity effects. The shedding frequencies, average drag coefficients as well as rms lift coefficient became almost similar for both cylinders in this flow pattern. The wakes of both cylinders were seen like a single square cylinder.
6. The mean pressure on both cylinders was found to vary irregularly in case of flip flopping flow pattern while for all other flow patterns case the pressure varied smoothly between cylinders.
7. At low G, the secondary cylinder interaction frequencies appeared frequently due to random fluctuations in CL and a strong jet flow effect.

## Data Availability

Data will be available on request by contacting the corresponding author, Dr. Ahmed Refaie Ali, via ahmed.refaie@science.menofia.edu.eg, OR via Dr. Afraz Hussain Majeed at afraz@ujs.edu.cn.

## Abbreviations

Re: Reynolds number
B: Blockage ratio
G: Gap ratio
CL: Lift coefficient
CD: Drag coefficient
St: Strouhal number
$$\alpha$$: Rotational speed
$$\text{s}$$: Diameter ratio
$${\text{C}}_{1}$$: First cylinder
$${\text{C}}_{2}$$: Second cylinder
$\text{m}$: Mass of particles
$$\text{c}$$: Velocity vector
$$\text{F}$$: External force
$$\Delta \text{t}$$: Time interval
$\text{n}$: Number of molecules
${\text{c}}_{\text{s}}$: Speed of sound
$$\text{g}$$: Distribution function
${\text{g}}_{\text{i}}^{\text{eq}}$: Equilibrium distribution function
$\text{L}$: Length of domain
$\text{H}$: Height of domain
$${\text{L}}_{\text{u}}$$: Upstream length
$${\text{L}}_{\text{d}}$$: Downstream length
$\text{d}$: Size of obstacles
Cd-mean: Mean drag coefficient
Cdm-1: Cd-mean of ${\text{C}}_{1}$
Cdm-2: Cd-mean of ${\text{C}}_{2}$
Cd-rms: Root-mean-square of drag coefficient
Cl-rms: Root-mean-square of lift coefficient
St-1: Strouhal number of ${\text{C}}_{1}$
St-2: Strouhal number of ${\text{C}}_{2}$
Cdrms-1: Root-mean-square value of CD of ${\text{C}}_{1}$
Cdrms-2: Root-mean-square value of CD of ${\text{C}}_{2}$
Clrms-1: Root-mean-square Value of CL of ${\text{C}}_{1}$
Clrms-2: Root-mean-square Value of CL of ${\text{C}}_{2}$
Cl-amp: Amplitude of lift coefficient
Clamp-1: Amplitude of CL of ${\text{C}}_{1}$
Clamp-2: Amplitude of CL of ${\text{C}}_{2}$

## List of abbreviations

LBM: Lattice Boltzmann method
QSF: Quasi steady flow
SF: Synchronized flow
SLR: Shear layer reattachment
SBB: Single bluff body
MS: Modulated synchronized
VM: Vortex Merging
AC: Alternating co-shedding
SRB: Single rotating bluff body
IR: Inverted rotation
FDF: Fully developed flow
FF: Flip flopping
FFT: Fast Fourier transform
